# Tamoxifen Treatment of Breast Cancer Cells: Impact on Hedgehog/GLI1 Signaling

**DOI:** 10.3390/ijms17030308

**Published:** 2016-02-27

**Authors:** Victoria E. Villegas, Milena Rondón-Lagos, Laura Annaratone, Isabella Castellano, Adriana Grismaldo, Anna Sapino, Peter G. Zaphiropoulos

**Affiliations:** 1 Department of Biosciences and Nutrition, Karolinska Institutet, Huddinge 14183, Sweden; peter.zaphiropoulos@ki.se; 2 Doctoral Program in Biomedical Sciences, Universidad del Rosario, Bogotá 11001000, Colombia; 3 Faculty of Natural Sciences and Mathematics, Universidad del Rosario, Bogotá 11001000, Colombia; srondonl@unito.it (M.R.-L.); mabel.grismaldo@urosario.edu.co (A.G.); 4 Department of Medical Sciences, University of Turin, Via Santena 7, 10126 Turin, Italy; laura.annaratone@unito.it (L.A.); isabella.castellano@unito.it (I.C.); anna.sapino@unito.it (A.S.); 5 Institute for Cancer Research and Treatment (IRCC), Strada Provinciale 142, 10060 Candiolo, Italy

**Keywords:** GLI1, TAM resistance, breast cancer, Hedgehog signaling, cellular proliferation

## Abstract

The selective estrogen receptor (ER) modulator tamoxifen (TAM) has become the standard therapy for the treatment of ER+ breast cancer patients. Despite the obvious benefits of TAM, a proportion of patients acquire resistance to treatment, and this is a significant clinical problem. Consequently, the identification of possible mechanisms involved in TAM-resistance should help the development of new therapeutic targets. In this study, we present *in vitro* data using a panel of different breast cancer cell lines and demonstrate the modulatory effect of TAM on cellular proliferation and expression of Hedgehog signaling components, including the terminal effector of the pathway, the transcription factor GLI1. A variable pattern of expression following TAM administration was observed, reflecting the distinctive properties of the ER+ and ER− cell lines analyzed. Remarkably, the TAM-induced increase in the proliferation of the ER+ ZR-75-1 and BT474 cells parallels a sustained upregulation of GLI1 expression and its translocation to the nucleus. These findings, implicating a TAM-GLI1 signaling cross-talk, could ultimately be exploited not only as a means for novel prognostication markers but also in efforts to effectively target breast cancer subtypes.

## 1. Introduction

Aberrant activation of the Hedgehog (HH) signaling pathway has been implicated in several cancers including breast cancer [1,2]. HH signal transduction initiates by the ligand Sonic Hedgehog (SHH) binding to its receptor Patched (PTCH), which acts, in it unliganded form, as an inhibitor of the activity of the transmembrane co-receptor Smoothened (SMO). Activated SMO releases GLI proteins (GLIoma-associated oncogene) from cytoplasmic sequestration, allowing the translocation of these zinc-finger transcription factors to the nucleus, where the transcriptional programs in response to pathway activation are executed [3,4]. Expression of GLI1, one of the three GLI factors in mammals and its translocation to the nucleus, are considered valuable markers of HH pathway activation [5]. Three isoforms of GLI1 with variable expression patterns are known: full-length GLI1 [6], N-terminal deletion variant (GLI1-∆N) identified by Shimokawa *et al.* (2008) [7] and truncated GLI1 (tGLI1) identified by Lo *et al.* (2009) [8]. Moreover, in 2012 studies by Ramaswamy *et al.* [1,9] have demonstrated that GLI1 is abnormally expressed in breast cancer tissues and cells, and this was associated with tamoxifen-resistance of the breast cancer cells.

Tamoxifen (TAM) is a selective estrogen receptor (ER) modulator, considered to be the first targeted cancer therapy. TAM is the most commonly used drug in routine clinical practice and represents the “gold standard” treatment for ER+ breast tumors [10,11,12]. The ER is expressed in 60%–70% of breast tumors; therefore, these are candidates for endocrine therapy. However, patients with similar prognostic factors at diagnosis can vary substantially in their treatment response, develop resistance and eventually die [12].

Identification of genes and genetic pathways responsive to TAM could provide the necessary framework for understanding the complex effects of this drug on target cells. This may allow a rationalization, at least in part, of the development of cellular resistance to TAM treatment. Additionally, a better understanding of the mechanisms involved in TAM resistance would help to identify novel molecular targets for treatment therapies and develop more accurate clinicopathological prognostic factors.

Here, we present *in vitro* data using a number of different breast cancer cell lines, demonstrating the modulatory effect of TAM on cellular proliferation and expression of HH signaling components, in particular GLI1. These findings reveal that GLI1 activation can be implicated in the growth and progression of breast cancer; however, the precise mechanism by which GL11 contributes to TAM resistance remains unclear.

## 2. Results

### 2.1. Proliferation Assays

**Figure 1:**
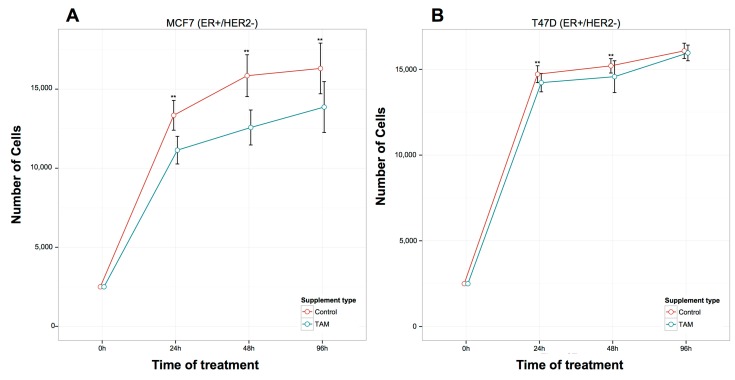

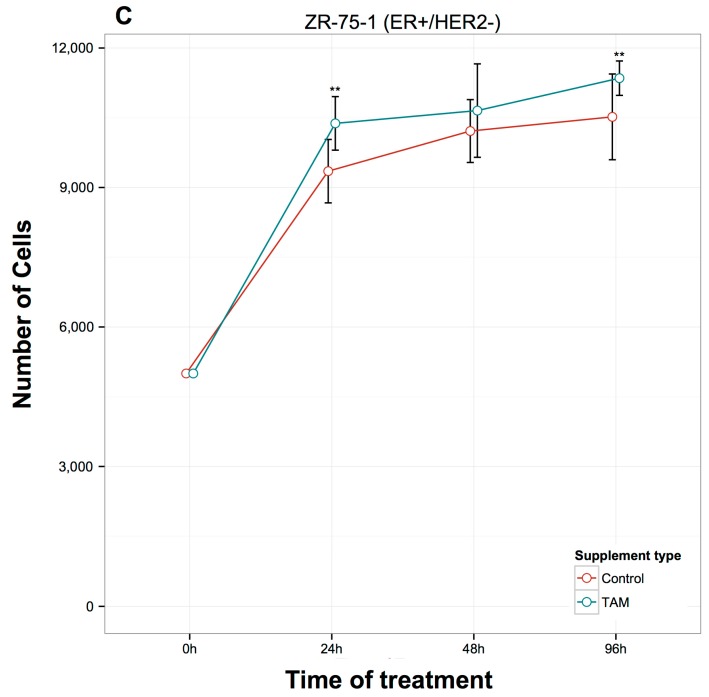
Effects of 1 μM TAM treatment for 24 h, 48 h, and 96 h on the proliferation of ER+/HER2-breast cancer cells. (**A**) MCF7; (**B**) T47D and (**C**) ZR-75-1. Error bars represent mean standard deviation of 16 separate experiments. Significant differences in treated *versus* untreated samples are indicated by the *p* values, ** *p* < 0.01.

**Figure 2:**
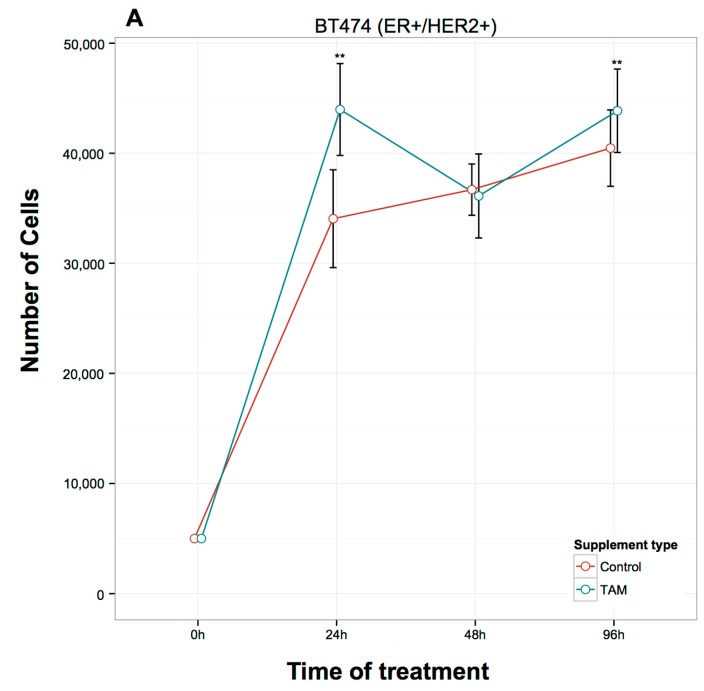

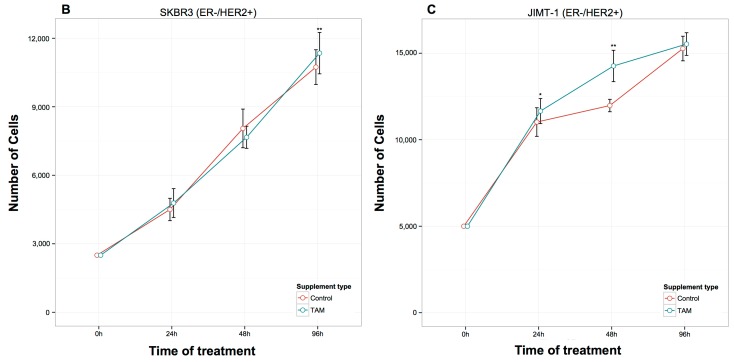
Effects of 1 μM TAM treatment for 24, 48, and 96 h on the proliferation of ER+/HER2+ (**A**) and ER-/HER2+ (**B**,**C**) breast cancer cells. (**A**) BT474; (**B**) SKBR3 and (**C**) JIMT-1. Error bars represent mean standard deviation of 16 separate experiments. Significant differences in treated *versus* untreated samples are indicated by the p values, * *p* < 0.05, ** *p* < 0.01.

TAM treatment significantly inhibited cell proliferation in MCF7 (ER+/HER2−) cells at 24, 48, and 96 h (Figure 1A), while in T47D (ER+/HER2−) cells, the inhibition of proliferation was not as pronounced, reaching significance only at 24 and 48 h (Figure 1B). In contrast to MCF7 and T47D cells, TAM induced a significant increase in the proliferation of ZR-75-1 (ER+/HER2−) cells at 24 h and 96 h after treatment (Figure 1C). Similar results in ZR-75-1 cells were also observed in BT474 (ER+/HER2+) cells: TAM induced a significant increase in the proliferation after 24 and 96 h of treatment (Figure 2A). Finally, in the ER-/HER2+ cell lines (Figure 2), the TAM effect was variable at different time points. In SKBR3 cells, TAM addition increased cellular proliferation at 96 h (Figure 2B), whereas, in JIMT-1 cells, TAM increased proliferation at 24 and 48 h (Figure 2C).

### 2.2. qRT–PCR Assays, GLI1 Variants, SMO and SHH Expression

**Figure 3:**
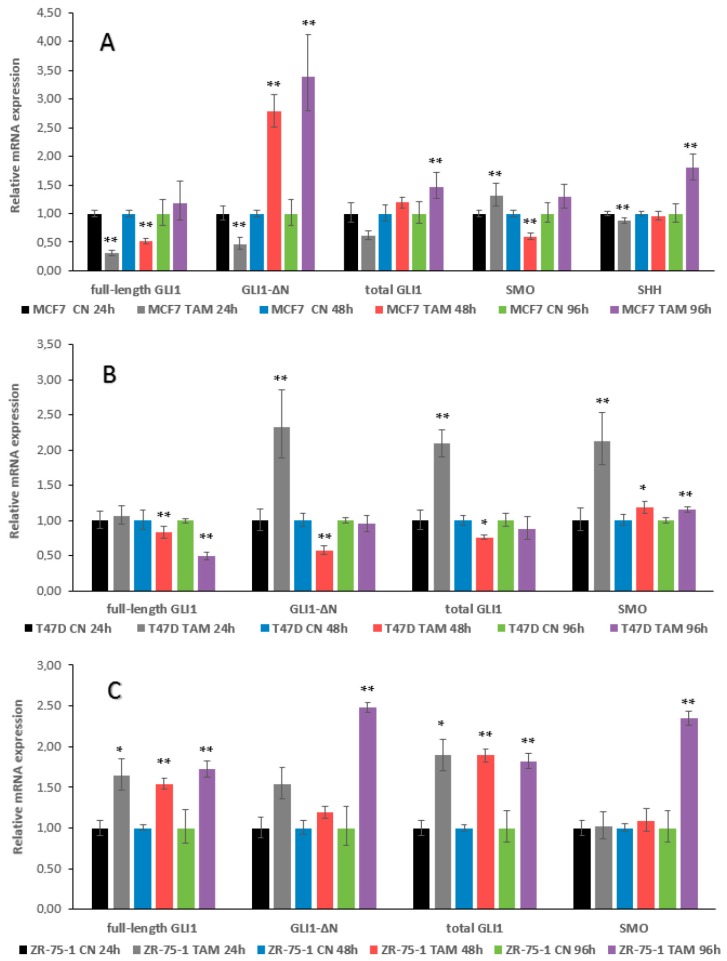
Effect of 1 μM TAM treatment for 24, 48, and 96 h on GLI1, SMO and SHH mRNA expression in ER+/HER2- breast cancer cells. (**A**) MCF7; (**B**) T47D; (**C**) ZR-75-1. CN, control cells; TAM, TAM-treated cells. Significant differences in treated *versus* untreated samples are indicated by the *p* values, * *p* < 0.05, ** *p* < 0.01.

TAM treatment of MCF7 cells for 24 h inhibited the mRNA expression of full-length GLI1, GLI1-∆N and SHH, while SMO expression was increased (Figure 3A). At 48 h, full-length GLI1 remained downregulated, in contrast to GLI1-∆N, which became upregulated. SMO also changed its pattern of expression and was downregulated. At 96 h, a tendency of increased expression in all genes tested was observed, which reached statistical significance for GLI1-∆N, total GLI1, and SHH (Figure 3A).

In T47D cells, no detectable expression of SHH was observed. Moreover, and in contrast to MCF7 cells, the expression levels of GLI1 were increased at 24 h, reaching significance for GLI1-∆N and total GLI1. However, at 48 and 96 h, there was a reversal of GLI1 expression, with this downregulation being most pronounced for full-length GLI1. SMO expression was significantly increased at all three time points (Figure 3B).

ZR-75-1 cells also had no detectable levels of SHH. However, in contrast to T47D and MCF7 cells, there was a sustained increase in GLI1 expression at 24, 48, and 96 h of TAM treatment, and this was particularly evident for full-length GLI1 and total GLI1. SMO expression did not change at 24 and 48 h but significantly increased at 96 h (Figure 3C).

**Figure 4:**
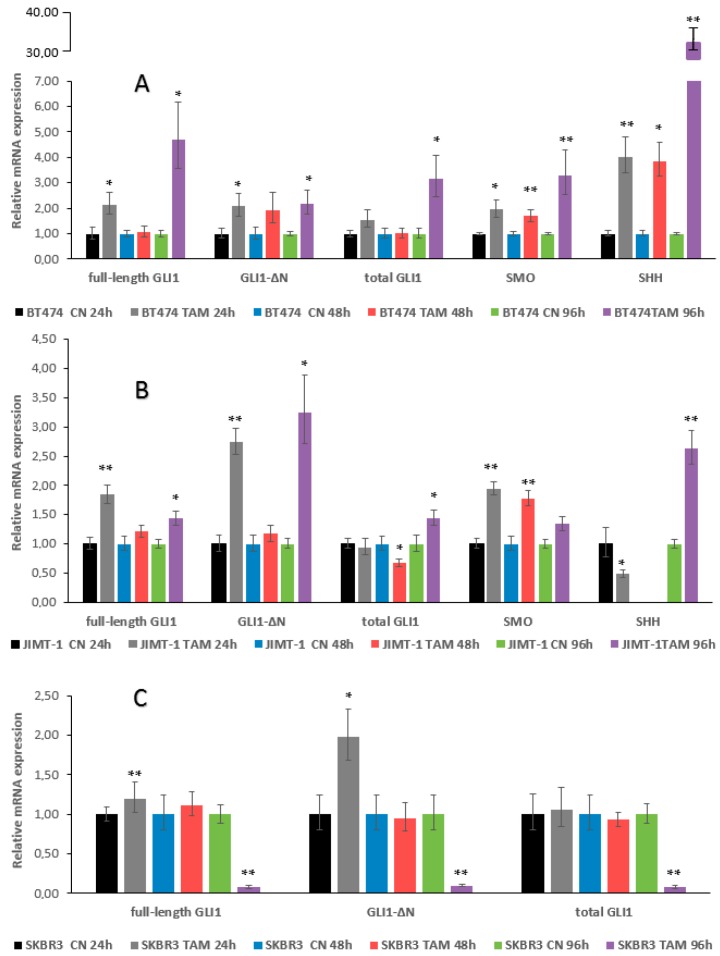
Effect of 1 μM TAM treatment for 24, 48, and 96 h on GLI1, SMO and SHH mRNA expression in ER+/HER2+ (**A**) and ER-/ HER2+ (**B**,**C**) breast cancer cells. (**A**) BT474; (**B**) SKBR3 and (**C**) JIMT-1. CN, control cells; TAM, TAM-treated cells. No data for SHH expression at 48 h in JIMT-1 cells are shown. Significant differences in treated *versus* untreated samples are indicated by the *p* values, * *p* < 0.05, ** *p* < 0.01.

BT474 cells were characterized by an increase in the expression of all genes tested at 24 h. At 48 h, there was a somewhat reduced increase; however, at 96 h, the observed increase reached statistically significance for all genes. It is worth noting the major increase in SHH expression at 96 h, with this high upregulation observed only in this cell line (Figure 4A).

JIMT-1 and SKBR3 cells demonstrated increased expression of full-length GLI1 and GLI1-∆N at 24 h. In JIMT-1 cells, GLI1 expression was reduced at 48 h, but re-appeared at 96 h. These cells were also characterized by increased expression of SMO at 24 and 48 h, and of SHH at 96 h (Figure 4B). In SKBR3 cells, a particularly pronounced reduction of GLI1 expression at 96 h was observed (Figure 4C).

In conclusion, our data on the mRNA expression indicate that the most sustained activation of GLI1 is seen with the ZR-75-1 cells, and the most sustained activation of SMO and SHH with the BT474 cells.

### 2.3. Immunohistochemistry Assays (IHC), Protein Expression of GLI1 in Nucleus and Cytoplasm

**Figure 5:**
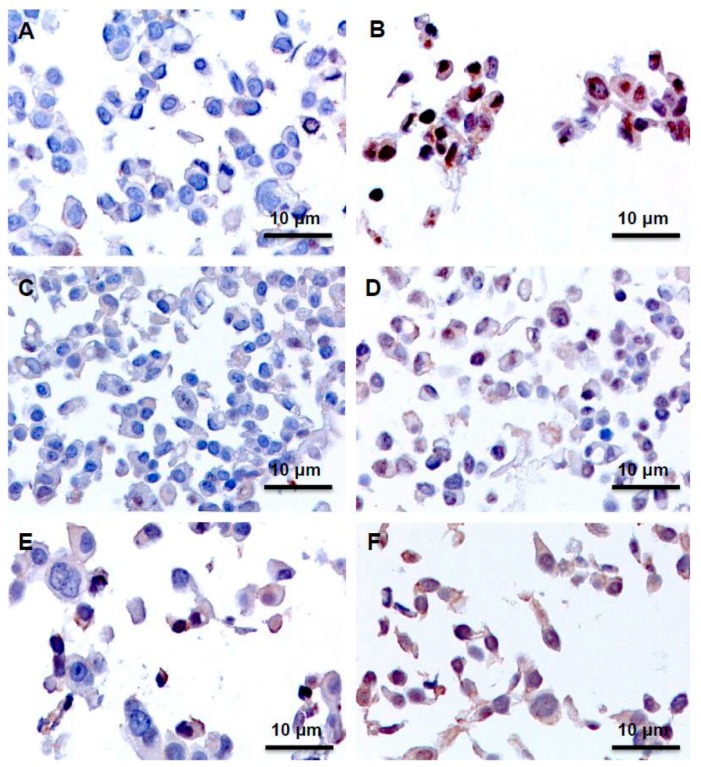
Immunohistochemical expression analysis of GLI1 protein in breast cancer cell lines without (**A**,**C**,**E**) or following 1 μM TAM treatment for 96 h (**B**,**D**,**F**). Representative pictures for GLI1 immunostaining showing negative expression in untreated MCF7 cells (**A**); positive expression in the nucleus and cytoplasm of MCF7 cells after TAM treatment (**B**); negative expression in untreated ZR-75-1 cells (**C**); positive expression in the nucleus of ZR-75-1 cells after TAM treatment (**D**); positive expression in untreated SKBR3 cells (**E**) and increased positive expression in the nucleus and cytoplasm of SKBR3 cells after TAM treatment (**F**). Scale bars: 10 μm.

As summarized in Table 1, the GLI1 protein expression detected by immunohistochemistry was variable among the different cell lines analyzed. All untreated cell lines, except T47D and SKBR3 cells, were negative for GLI1 expression (Figure 5).

Interestingly, BT474 and ZR-75-1 cells were the only ones that had a similar pattern of GLI1 expression. In these cells, GLI1 expression was clearly detectable in the nucleus following 24, 48, and 96 h of TAM treatment (Figure 5).

In MCF7 and SKBR3 cells lines administration of TAM resulted in cytoplasmic and nuclear signals at all three time points (Figure 5).

In T47D cells GLI1 expression was increased at 24 h. However, this was not sustained, as no signals were observed at 48 and 96 h.

In JIMT-1 cells no GLI1 expression was detected in untreated or after 24 and 48 h of TAM administration. Nuclear and cytoplasmic signals were, however, observed at 96 h.

In conclusion, the cells that most clearly show a nuclear translocation of GLI1, an event characterizing activation of Hedgehog signaling, are ZR-75-1 and BT474, the same cells that exhibited the most sustained increases in the mRNA expression of Hedgehog signaling components.

## 3. Discussion

In this study, the impact of TAM administration on breast cancer cell lines was investigated. Specifically, the role of canonical, SMO-/SHH-dependent HH signaling [13,14] and non-canonical, SMO-/SHH-independent HH signaling [15,16] was addressed, as this pathway has been linked to tumorigenesis including breast cancer [17,18,19]. Additionally, the best-known marker of HH signaling activation, the transcription factor GLI1, was interrogated by expression analysis of full length GLI1 and the GLI1-∆N variant. Expression of the tGLI1 variant was too low for accurate determinations.

Our results show that cell lines that express nuclear GLI1 staining after TAM treatment exhibit an increase in cell proliferation compared to control, GLI1 negative cells. These findings could indicate that the HH signaling pathway is an alternative growth promoting mechanism that can be activated by TAM in breast cancer cells. In fact, it has recently been reported that activation of this pathway facilitates tumor growth and progression, supporting an association of HH signaling with increased risk of metastasis and breast cancer-specific death [20]. In this context, it is worth mentioning that the increased risk of breast cancer by vitamin D deficiency may be, at least partly, mediated via HH signaling, since vitamin D acts as an antagonist of this signaling pathway [21,22].

Here, the detailed analysis of breast cancer cells provides evidence that TAM treatment reduces the proliferation of the ER+/HER2− cell lines MCF7 and T47D. In MCF7 cells, there is a tendency towards a reduction in the mRNA expression of GLI1 and SHH at 24 h, but at 96 h an increased expression was observed. The immunohistochemical data on GLI1 are in-line with the increased mRNA expression at 96 h. T47D cells express no SHH, with SMO and GLI1 expression increasing at 24 h, then GLI1 expression drops at 48 and 96 h, which is indicative of a strong correlation with the immunohistochemical data.

The ER+/HER2− ZR-75-1 and the ER+/HER2+ BT474 cell lines show similar proliferation patterns, namely, an increase following TAM administration, in addition to comparable mRNA and protein expression. Significantly, in both cell lines the immunohistochemical data demonstrate a TAM-dependent expression of GLI1 in the nucleus. Compared to MCF7 and T47D these two cell lines have a more aggressive profile, and this is in-line with the 2012 report by Li *et al.* [9], where nuclear GLI1 expression was correlated with unfavorable prognosis in breast cancer.

Interestingly, previous studies in breast cancer have demonstrated that nuclear staining of GLI1 in cancer cells is associated with aggressive tumor characteristics (*i.e.*, tumor stage and lymph node status) [18], early recurrence, and poor prognosis [23,24]. In addition, our results could be indicative not only of an aggressive cellular state following GLI1 nuclear translocation after TAM addition, but also may have implications on the possible role of GLI1 in TAM resistance therapy.

In the ER−/HER+ SKBR3 cell line, the immunohistochemical data show an increase in the expression of GLI1 following TAM treatment, with this correlating to the mRNA expression only at 24 h. The cellular proliferation does not change until 96 h, where TAM elicits an increase. Interestingly, at this time-point, a pronounced reduction of GLI1 mRNA expression is observed. The other ER−/HER+ cell line, JIMT-1, also increased its proliferation by TAM, but at 24 and 48 h. A correlation of higher GLI1 mRNA expression and protein detection by immunohistochemistry was observed at 96 h of TAM treatment.

Although the SKBR3 cell line is negative for ER, this expresses the novel functional estrogen transmembrane receptor G protein-coupled receptor 30 (GPR30) [25]. Consequently, the observed effect of TAM on HH signaling pathway could be attributed to this receptor. Biological effects mediated by GPR30 are maintained when classic ERs are absent or blocked [26]. Furthermore, activation of GPR30 signaling in response to TAM and a subsequent increase in cell proliferation in breast cancer cells have also been observed [27,28,29].

In conclusion, we find that the TAM-dependent increase in the proliferation of the ER+ ZR-75-1 and BT474 cells is in-line with increased GLI1 expression in the nucleus. In the ER− SKBR3 and JIMT-1 cell lines, the observed pattern is different. Increased proliferation appears to relate with decreased GLI1 expression, as assessed by combining immunohistochemical and mRNA detection. For the ER+ MCF7 and T47D cells, there is no consistent correlation of GLI1 expression and proliferation changes. Worth noting is the lack of detectable SHH expression in ZR-75-1 and T47D cells, with this observation implicating non-canonical HH signaling in these two cellular settings.

Although the MCF7 cell line is the most studied in the determination of the gene expression profiles in response to treatment with TAM [30,31], the variable pattern of expression of the TAM-regulated genes examined in our study, both in ER+ and ER− cells, is likely to reflect the distinctive properties of these cells. It is therefore conceivable that these findings could, ultimately, be exploited not only to identify the response of various breast cancer cell types to TAM, but also to examine whether GLI1 expression may serve as marker of TAM sensitivity and/or resistance.

Overall, our findings suggest that GLI1 may represent a new important prognostic marker in breast cancer, thereby supporting the use of combined therapies involving HH pathway inhibitors and endocrine treatment in breast cancer. It should also be mentioned that therapeutic targeting of HH signaling using inhibitors acting on SMO has resulted in limited success, due to the development of resistance. However, the current efforts to use inhibitors that act further downstream of the pathway, at the level of the GLI factors, are promising, as these would also block non-canonical signaling [32].

## 4. Materials and Methods

### 4.1. Cell Lines

The human breast cancer cell lines MCF7, T47D, ZR-75-1 (ER+/HER2−), BT474 (ER+/HER2+), SKBR3 and JIMT-1 (ER−/HER2+) were obtained from the American Type Culture Collection (ATCC, Manassas, VA, USA). The MCF7, T47D, ZR-75-1, SKBR3 and JIMT-1 cell lines were cultured in an RPMI 1640 medium (Sigma, St. Louis, MO, USA), while the BT474 cell line was cultured in a DMEM medium (Sigma). All culture media were supplemented with 10% fetal bovine serum (FBS) (Sigma), an antibiotic-antimycotic solution (1×) (Sigma), and l-glutamine (2 mM) (Invitrogen GmbH, Karslruhe, Germany). The cultures were maintained in an incubator at 37 °C under 5% CO_2_.

### 4.2. Treatment of the Cell Lines with TAM

In order to remove endogenous serum steroids and eliminate the weak estrogen agonistic activity of phenol red, 48 h prior to the addition of TAM (T5648, Sigma), cells were washed with 5 mL of phosphate buffered saline (PBS), and were then switched to phenol red-free RPMI 1640 (Sigma) containing 10% charcoal-stripped fetal bovine serum (FBS) (Sigma). TAM was dissolved in absolute ethanol, and diluted in the media at 1 µM, and was then added to the culture medium for 24, 48, and 96 h. Cells without TAM treatment were used as controls.

### 4.3. Proliferation Assays

Cells were seeded at a density of 2.5–5 × 10^3^ cells per 100 µL of phenol red-free medium in a 96-well multi-well plate. After 24 h, cells were treated with 1 µM TAM for 24, 48, and 96 h; controls were cells without TAM treatment. At the end of each treatment, cell viability was assessed using the cell proliferation ELISA kit, BrdU (Roche Diagnostics Deutschland GmbH, Mannheim, Germany). Measurements of absorbance were performed using a MultiSkan Bichromatic reader (Labsystems, Helsinki, Finland) against a background control as blank. Experiments were performed in 16 replicates and expressed as means ± SD.

### 4.4. RNA Isolation

RNA from control and treated cells was prepared with the RNeasy kit (Qiagen, Hamburg, Germany) followed by verification of the purity and integrity using the ratio of absorbance at 260 nm and 280 nm. cDNA synthesis was carried out with Fermentas First Strand cDNA Synthesis Kit for RT-PCR.

### 4.5. Real-Time PCR

Real-time PCR was carried out with Roche Enzyme Fast start Universal SYBR Green Master ROX on a 7500 fast real-time PCR system (Applied Biosystems, Foster, CA, USA) and LightCycler^®^ 96 System (Roche Diagnostics GmbH, Mannheim, Germany) with primers designed to detect full-length GLI1 and GLI1-∆N variant, total GLI1, SHH, and SMO. The housekeeping gene TBP was used as an internal control based on the least variation of *C*_t_ values in the samples analyzed. Quantification of the relative expression of target genes was performed using the ΔΔ*C*_t_ method (fold difference 2^−ΔΔ*C*t^). The following PCR primers were used: TBP-E3 (exon 3) forward 5′-GCCAGCTTCGGAGAGTTCTGGGATT and E4 reverse 5′-CGGGCACGAAGTGCAATGGTCTTTA; full-length GLI1-E3 forward 5′-CCACAGTTATGGGCCAGCCAGAGAG, GLI1-∆N-E1/4 forward 5′-GCGCCCAGACAGAGGCCCACT and E4 reverse for both GLI1 variants 5′-GGCATCCGACAGAGGTGAGATGGAC; total GLI1-E11 forward 5′-CAGCTACATCAACTCCGGCCAATAGGG and E12 reverse 5′-TGCTGCGGCGTTCAAGAGAGA CTG; SMO-E11 forward 5′-TTTCTGTCACCCCTGTGGCAACTCC and E12 reverse 5′-CGGGCACACCTCCTTCTTCCTCTTC; SHH-E1 forward 5′-GATGAAGAAAACACCGGAGCGGACA and E2 reverse 5′-TCCTCTGAGTGGTGGCCATCTTCGT.

### 4.6. Immunohistochemistry Assays (IHC)

Immunohistochemistry detection for GLI1 was carried out on Formalin-Fixed Paraffin Embedded (FFPE) cell blocks from each cell line (MCF7, T47D, ZR-75-1, BT474, SKBR3 and JIMT-1) with or without 1 µM TAM for 24, 48, and 96 h. At the indicated time points, cells were fixed in formalin, included in paraffin, and IHC analyses were performed using the rabbit polyclonal primary antibody GLI1 (1:600 dilution; Novus, CO, USA). GLI1 expression was evaluated by determining the percentage of cells exhibiting immunoreaction. Cells sections in which >10% of cells were stained were considered to be positive. GLI1 immunoreactivity was compared in control *versus* treated cell lines. Immunostaining evaluation was performed independently by three blinded investigators. Positive values given as Positive+ were used to indicate a higher percentage of cells expressing GLI1 after the addition of TAM compared to control.

### 4.7. Statistical Analysis

The Student’s *t*-test was performed to compare cell proliferation and relative expression from treated and untreated cell lines. *p* values less than 0.05 were considered significant (* *p* < 0.05, ** *p* < 0.01).

## Figures and Tables

**Table 1:** Immunohistochemical expression analysis of GLI1 protein in breast cancer cell lines without (Control) or following 1 μM TAM treatment. Quantification and assessment of positivity were performed as described in Materials and Methods.

Cell Line	Control	TAM 24 h	TAM 48 h	TAM 96 h
**MCF7**	Negative	Positive (Nucleus and Cytoplasm)	Positive (Nucleus and Cytoplasm)	Positive (Nucleus and Cytoplasm)
**T47D**	Positive	Positive + (Nucleus and Cytoplasm)	Negative	Negative
**ZR-75-1**	Negative	Positive (Nucleus)	Positive (Nucleus)	Positive (Nucleus)
**BT474**	Negative	Positive (Nucleus)	Positive (Nucleus)	Positive (Nucleus)
**SKBR3**	Positive	Positive + (Nucleus and Cytoplasm)	Positive + (Nucleus and Cytoplasm)	Positive + (Nucleus and Cytoplasm)
**JIMT-1**	Negative	Negative	Negative	Positive + (Nucleus and Cytoplasm)

## Supplementary Materials

Supplementary materials can be found at http://www.mdpi.com/1422-0067/17/3/308/s1.

## Abbreviations

ER: estrogen receptor; GLI1: Glioma-associated oncogene 1; GLI: Glioma-associated oncogene; GLI1-∆N: N-terminal deletion GLI1 variant; HER2: human epidermal growth factor receptor 2; HH: Hedgehog; SHH: Sonic Hedgehog; SMO: transmembrane co-receptor Smoothened; TAM: tamoxifen; TBP: TATA-binding protein.
