# Supplementary material for: Tamoxifen Treatment of Breast Cancer Cells: Impact on Hedgehog/GLI1 Signaling
Source: Int J Mol Sci. 2016 Feb 27;17(3):308. doi: 10.3390/ijms17030308 (PMC4813171; doi:10.3390/ijms17030308)
Supplement: Supplementary file 1 [file ijms-17-00308-s001.docx]

Tamoxifen Treatment of Breast Cancer Cells: Impact on Hedgehog/GLI1 Signaling

Victoria E. Villegas, Milena Rondón-Lagos, Laura Annaratone, Isabella Castellano,
Adriana Grismaldo, Anna Sapino and Peter G. Zaphiropoulos


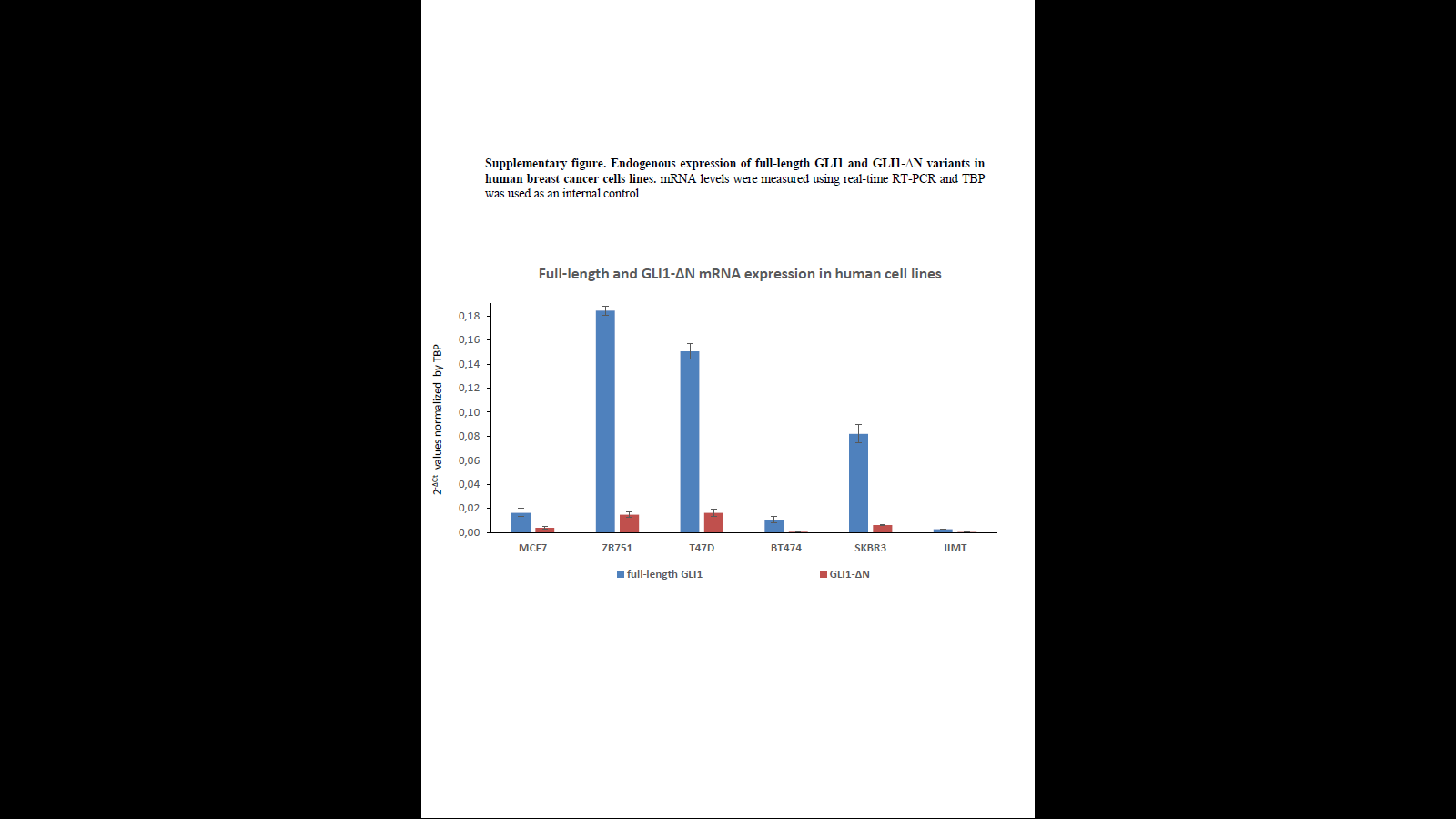


**Figure S1.** Endogenous expression of full-length GLI1 and GLI1-ΔN variants in human breast cancer cells lines. mRNA levels were measured using real-time RT-PCR and TBP was used as an internal control.
